# The role of targeting glucose metabolism in chondrocytes in the pathogenesis and therapeutic mechanisms of osteoarthritis: a narrative review

**DOI:** 10.3389/fendo.2024.1319827

**Published:** 2024-03-06

**Authors:** Peng Pi, Liqing Zeng, Zhipeng Zeng, Keqiang Zong, Bing Han, Xizhe Bai, Yan Wang

**Affiliations:** ^1^ School of Sports Medicine and Rehabilitation, Beijing Sport University, Beijing, China; ^2^ School of Physical Education, Qiqihar University, Heilongjiang, Qiqihar, China; ^3^ College of Physical Education and Health, East China Normal University, Shanghai, China

**Keywords:** osteoarthritis, glucose metabolism, cartilage, glycolysis, therapeutic

## Abstract

Osteoarthritis (OA) is a common degenerative joint disease that can affect almost any joint, mainly resulting in joint dysfunction and pain. Worldwide, OA affects more than 240 million people and is one of the leading causes of activity limitation in adults. However, the pathogenesis of OA remains elusive, resulting in the lack of well-established clinical treatment strategies. Recently, energy metabolism alterations have provided new insights into the pathogenesis of OA. Accumulating evidence indicates that glucose metabolism plays a key role in maintaining cartilage homeostasis. Disorders of glucose metabolism can lead to chondrocyte hypertrophy and extracellular matrix degradation, and promote the occurrence and development of OA. This article systematically summarizes the regulatory effects of different enzymes and factors related to glucose metabolism in OA, as well as the mechanism and potential of various substances in the treatment of OA by affecting glucose metabolism. This provides a theoretical basis for a better understanding of the mechanism of OA progression and the development of optimal prevention and treatment strategies.

## 1 Introduction

Articular cartilage (AC) is a complex connective tissue, which is responsible for bearing, shock absorption and lubrication of joints of the whole body (1). Chondrocyte is the main cell type in normal human cartilage, which is differentiated from mesenchymal cells and accounts for 1-5% of cartilage tissue (2). Chondrocytes are surrounded by the pericellular matrix (PCM), which is considered a buffer of physical forces between chondrocytes and the extracellular matrix (ECM) (3). When chondrocytes are subjected to abnormal mechanical stimulation, such as overloading or joint injury, their metabolic balance is altered, leading to matrix loss and tissue degeneration, which can lead to osteoarthritis (OA) (4, 5). OA is one of the most common and diverse degenerative diseases with complex pathogenesis that affects all joint tissues, characterized by cartilage degeneration, osteophyte formation, meniscal degeneration, subchondral bone remodeling, joint inflammation and fibrosis of the infrapatellar fat pad (6–12). The main clinical manifestations of OA patients are AC degeneration, subchondral bone sclerosis, synovitis, osteophyte formation, joint pain and disability (13). Globally, OA affects 10% - 12% of the adult population and this number is expected to increase rapidly over the next two decades (6). Although many risk factors are known to contribute to OA, including aging, obesity, genetics, joint biomechanics, and previous joint damage, the pathogenesis of OA remains unclear (14, 15). Due to the difficulty of cartilage recovery after damage, at present, there is no other more effective treatment except the use of analgesic drugs and the selection of surgical joint replacement as the final treatment option for OA (16, 17). Considering the huge population affected by OA, it not only affects the quality of life of patients, but also imposes a huge economic burden on society (7). It is crucial to investigate the pathogenesis of OA and explore new therapeutic approaches.

Energy metabolism is an important regulator of cellular function (18). Under adverse conditions, most mammalian cells undergo an energy metabolic transition (i.e., switching from a static regulatory state to an active metabolic state) to maintain energy homeostasis and promote cell survival (19–21). It has been shown that energy metabolism is critical for cartilage homeostasis and is drastically altered in chondrocytes of OA cartilage (16). In addition, recent studies have shown that chondrocytes can undergo metabolic alterations in response to different stimuli (18, 22). Normally, chondrocytes *in vivo* tend to produce ATP through glycolysis rather than oxidative phosphorylation (OXPHOS) due to the relatively hypoxic environment and maintain a stable ECM by balancing anabolic and catabolic processes (16). However, the TCA cycle and electron transport chain (ETC) efficiently produce ATP in the presence of oxygen through OXPHOS (23). Furthermore, stressed cells, such as immune cells and cancer cells, are capable of generating ATP by aerobic glycolysis when oxygen is available (18). Such cellular metabolic alterations are not only important for the maintenance of energy balance, but probably also critical for alterations in cellular function and signaling (18). Hence, regulation of metabolism may play a vital role in the treatment of OA.

As the main content of energy metabolism, lipid metabolism, amino acid (AA) metabolism and glucose metabolism play a crucial role in the pathogenesis of OA (21). The pathogenesis of OA involves a variety of lipid metabolic changes in cartilage, subchondral bone, and periosteum, which interact with inflammatory mediators to affect the development of lesions (24). Furthermore, some adipokines can directly regulate inflammation and promote the development of OA, thereby affecting joint health (25). Since a variety of AA have been found to be abnormally expressed in chondrocytes, it has also been proposed that the establishment and development of OA are related to the alterations in AA metabolism and profiles, including glutamate- and arginine-family AA as well as their related metabolites (26). Additionally, increasing evidence has shown that glucose metabolism plays a key role in maintaining cartilage homeostasis. Disorders of glucose metabolism can lead to chondrocyte hypertrophy and ECM degradation, contributing to the development of OA (27). It is suggested that targeting enzymes and factors affecting glucose metabolism may be a major breakthrough in the treatment of OA.

At present, many reviews have summarized the role of lipid metabolism and AA metabolism in the pathogenesis of OA in detail. However, no article has specifically targeted a comprehensive summary of the association between glucose metabolism and OA. This narrative review focused on the research literature related to glucose metabolism and OA, aiming to summarize the potential mechanisms of glucose metabolism in the regulation of OA, and provide a reference for further research on the role of glucose metabolism in the treatment of OA and other related diseases. We used “osteoarthritis,” “glucose,” “metabolism,” “mechanism” and “therapeutic” as keywords. Then we searched PubMed, Embase and Web of science for methodological papers on articles from inception to September 2023, especially recently 5 years. Inclusion criteria included: (a) research on OA and its related diseases, (b) the experimental subjects were humans or animals, (c) targeting proteins or factors related to glucose metabolism. Exclusion criteria included: (a) article was not written in English, (b) the full-text was not available or published, (c) conference papers, (d) reviews or meta-analysis. A total of 950 relevant articles were retrieved, and 81 were finally selected for this review.

## 2 An overview of glucose metabolism

Glucose plays a crucial role in cartilage development, stability, repair, and remodeling (19). Like most cells, chondrocytes use glucose metabolism to produce ATP as their primary energy source, and at the same time, also require glucose as a structural precursor for glycosaminoglycan synthesis (28). There are three major pathways involved in glucose metabolism: glycolysis, pentose phosphate pathway, and tricarboxylic acid cycle (28–30). Among them, glycolysis is tightly regulated as it is required to provide energy for cell survival and specific cellular functions such as inflammation (31). Dysregulation or inhibition of any pathway can lead to lactic acidosis and/or energy deficit, ultimately resulting in abnormal cellular processes that contribute to the formation of several chronic diseases (31). Notably, abnormal glucose metabolism dysregulation has been demonstrated in human cancers as well as abnormal vascular smooth muscle cells, endothelial cells, and human rheumatoid arthritis (RA) chondrocytes, suggesting that glucose metabolism disorders are associated with a variety of diseases (32).

OA is now considered to be a metabolism-related disease, and its establishment and development are closely related to inflammation and metabolism (33). In OA, low-grade inflammation leads to chondrocyte hypoxia, which changes energy metabolism from a resting state to an active state (34). At this time, as the primary energy source for chondrocytes, glycolysis increases to meet the energy demand of chondrocytes (35). Specifically, unlike many tissues, AC has no blood, nerves, or lymphatic vessels, and the synovial fluid in the joint is its source of nutrients (36). Therefore, oxygen and glucose are more difficult to obtain compared to plasma. Its relatively hypoxic environment results in lower energy production by OXPHOS (27). Since glycolysis is a process of rapid ATP generation, chondrocytes are extremely dependent on glycolysis for energy production. However, excessive enhancement of glycolysis also leads to decreased energy acquisition, which inhibits cell proliferation and differentiation. Disturbance of glycolytic metabolism can lead to chondrocyte hypertrophy and ECM degradation (27, 37). Accumulating evidence indicated that glucose metabolism disorders are the cause of OA. Among them, different enzymes and factors play an important role in the process of glucose metabolism, and may also be involved in the pathogenesis of OA. Targeting glucose metabolism is expected to make a major breakthrough in the pathogenesis and treatment of OA. Therefore, in the following few sections, we summarized the potential targets such as related enzymes and factors involved in glucose metabolism in OA as well as therapeutic substances, respectively.

## 3 Role of glucose metabolism mediated by different enzymes in OA

### 3.1 Role of GLUT1-mediated glucose metabolism in OA

Since glucose is a large molecule, its diffusion across a membrane is difficult. Hence, in most cell types, glucose is transported across the plasma membrane through facilitated diffusion along a concentration gradient and does not require energy (38, 39). Glucose metabolism in normal AC has been demonstrated to respond to anabolic and catabolic signals by regulating the expression of multiple facilitative glucose transporters of the Glut family, such as glucose transporter type 1 (GLUT1). GLUT1 is up-regulated under hypoxic and glucose-deprived conditions to enhance glucose uptake and decreased under high-glucose conditions to balance glucose levels in chondrocytes (40). Earlier studies *in vitro* showed that the loss of *GLUT1* gene leads to AC cytopenia and proteoglycan loss in mice, indicating that GLUT1-mediated glucose metabolism is required for chondrocyte growth (41). Recent studies have found in mice that genetic defects in chondrocyte GLUT1 during development cause chondrodysplasia, which may result in structural and mechanical abnormalities of AC and lead to OA in adulthood (41–43). In addition, Li et al. (44) revealed that GLUT1 programming changes play an important role in increased AC matrix degradation and susceptibility to OA in female prenatal caffeine exposure (PCE) offspring rats. At the same time, they also established a PCE-induced intrauterine growth retardation (IUGR) model in rats to elucidate the role of altered glucose transport function mediated by altered GC- insulin-like growth factor-1 (IGF1)-GLUT1 axis programming in the development of fetal-originated OA and its epigenetic mechanism, providing a theoretical basis for the early prevention and treatment of OA. Furthermore, using genetic and metabolic pathways, Wang et al. (28) found in mice that GLUT1-mediated glucose metabolism is required for normal growth plate and showed that GLUT1 loss-of-function (LOF) causes GP cartilage and AC abnormalities. The latest evidence found in mouse suggests that forced expression of GLUT1 in AC is able to prevent OA progression. Notably, *in vitro* studies of human OA chondrocytes, the sustained elevation of GLUT1 expression can increase glucose uptake and produce excess advanced glycation end products (AGEs), which degrade cartilage (45). Moreover, Pfander et al. (46) found in human OA AC that the amount of GLUT1 increased with the severity of OA. A study of human OA chondrocytes reported that although OA chondrocytes were able to adapt to glucose deprivation, high glucose exposure failed to down-regulate GLUT-1 expression. This may be a crucial pathogenic mechanism by which diseases characterized by hyperglycemia promote degenerative changes in chondrocytes, thereby contributing to the progression of OA (47).

Overall, the GLUT1 transport function is essential for glucose uptake in cartilage. Targeting GLUT1 to regulate glucose metabolism may alter chondrocyte maturation processes, including cell proliferation, hypertrophy and cartilage matrix production, suggesting that GLUT1-mediated glucose metabolism plays a key role in cartilage ossification and development. Therefore, GLUT1 may be a potential target for the treatment of OA. However, the exact mechanisms by which glucose metabolism maintains AC health may be multifaceted and warrant further investigation.

### 3.2 Role of HK2-mediated glucose metabolism in OA

Hexokinases (HKs) catalyze the first step of glucose metabolism, that is, the conversion of glucose to glucose-6-phosphate (G6P) (48). Hexokinase 2 (HK2), an isoform of HK, has a high affinity for glucose compared with other enzymes. Notably, to maintain a concentration gradient, HK2 initiates major glucose utilization pathways such as glycolysis, pentose phosphate pathway, and OXPHOS while promoting glucose entry into the cell (29, 49). Recently, Bustamante et al. (50) explored the role of HK2 in bone and cartilage injury, and found that overexpression of HK2 can lead to increased RNA expression levels of proinflammatory cytokines such as interleukin (IL)-6 and IL-8 in human OA fibroblast-like synoviocytes (FLS, a heterogenous cell population of the synovium that produces certain components of the synovial fluid and AC), suggesting a potential therapeutic effect of HK2 on OA and may be safer than overall glycolysis inhibition (51). Additionally, Wu et al. (31) investigated the energy metabolism of human primary chondrocytes in OA, as well as the role of glycolysis on AC under inflammatory conditions and found that gene expression of *HK2* was increased in severe OA cartilage. Although no significant changes in HK2 were found at the overall protein level, an increase in G6P was found at the metabolomic level of cartilage collected from severely injured region, indicating an elevated level of HK2 activity in OA.

In general, HK2 plays an important role in cartilage glycolysis by regulating cellular metabolism, which greatly expands our understanding of glucose metabolism in the pathogenesis of OA. New treatments for OA or related alternative outcomes may be developed in the future, especially small-molecule drugs that target HK2 downstream proteins or kinases.

### 3.3 Role of LDHA-mediated glucose metabolism in OA

Lactate dehydrogenase A (LDHA), the reduced form of nicotinamide-adenine dinucleotide (NADH), is one of the key enzymes in aerobic glycolysis, which is mainly used to catalyze the last step of glycolysis, i.e. the conversion of pyruvate and finally into lactate via the alternative oxidation of the cofactor, from reduced form of NADH to oxidized form (NAD+) (32, 52). In the normoxic environment, glucose is catalyzed to produce pyruvate, which then enters the mitochondria and undergoes OXPHOS through the TCA cycle. Conversely, under a hypoxic state, high-throughput glycolysis occurs primarily in the cytoplasm via LDHA (32). Previous studies have reported that LDHA is highly expressed in aerobic glycolytic cancer lineages, suggesting that the up-regulation of LDHA can be induced in a hypoxic state (53). In addition, the aberrant expression of LDHA may be an important initiator of RA joints (54, 55). A recent study explored the effect of glycolytic inhibition on hyaluronic acid synthetase 2 (HAS2) synthesis and inflammation development in synovial fibroblasts (SFs) from TMJOA patients and found that the use of a specific inhibitor of LDHA inhibited lactate secretion and promoted HAS2 and hyaluronic acid (HA) synthesis in temporomandibular joint OA (TMJOA) SFs (32). HA is an important matrix component in cartilage. High molecular weight HA can participate in the anti-inflammatory process by forming a protective coating of chondrocytes (56). Interestingly, recent *in vivo* results in a rat OA model showed that the combination of HA and lactose-modified chitosan (Chitlac; CTL) inhibited cartilage degradation more than HA alone (57). This provides a new therapeutic approach for OA. Furthermore, Arra et al. (18) have shown in studies of mouse and human OA chondrocytes that a metabolic shift in cellular metabolism toward glycolysis and LDHA reprogramming may occur in chondrocytes under inflammatory conditions. Moreover, they also found a pro-reactive oxygen species (ROS) formation function of LDHA in chondrocytes under inflammatory states (18). ROS are considered to be one of the potential factors leading to OA disease due to their ability to modify proteins and lipids, damage DNA and other cellular adverse reactions (58–61). Although ROS play an important signaling function under normal physiological conditions, elevated ROS as a mediator of disease progression in OA is considered to carry significant pathological risks (62). Besides, it has been specifically reported that upregulation of pyruvate kinase M2 (PKM2) and LDHA expression was found in OA chondrocytes compared to healthy controls, indicating an increased activity of glycolysis (18, 63). This suggests a shift from anti-inflammatory dependent OXPHOS and TCA cycle to pro-inflammatory dependent glycolytic energy in OA chondrocytes, identifying LDHA as a potential target for OA therapy.

In summary, LDHA is a key glycolytic enzyme that promotes the conversion of pyruvate to lactate and ROS production in chondrocytes under inflammatory conditions. Since there are many pathways for ROS formation, further studies are needed to prove its source. Previous studies have shown that LDHA plays an important role in the development of RA as well as TMJOA, suggesting the potential of LDHA in the treatment of OA.

### 3.4 Role of PKM2-mediated glucose metabolism in OA

PKM2 is one of the major rate-limiting enzymes in glycolysis and plays an important role in the regulation of glycolysis by catalyzing phosphoenolpyruvate (PEP) to pyruvate and ATP (63). Yang et al. (63) investigated the involvement of PKM2 in the pathogenesis of OA by exploring its role in glycolysis and collagen matrix production in OA chondrocytes. PKM2 expression has been reported to be upregulated in isolated OA chondrocytes compared to healthy control chondrocytes. In addition, glucose consumption and lactate production were significantly inhibited in the PKM2 knockout group, while GLUT1, LDHA and hypoxia-inducible factor 1α (HIF-1α) were decreased, suggesting that PKM2 knockout inhibited glycolysis in OA chondrocytes. Furthermore, the hydrolysis of cartilage ECM proteins is the main pathogenesis of OA, and *PKM2* gene knockout reduces the production of extracellular collagen matrix, which in turn leads to the degeneration of AC in OA. Interestingly, when PKM2 expression is upregulated in OA chondrocytes, lactate accumulates under the catalysis of LDHA, forming an acidic environment that inhibits chondrocyte matrix synthesis and possibly promotes OA cartilage degeneration (64, 65).

In conclusion, PKM2 expression and activity were increased in OA chondrocytes, while PKM2 knockdown inhibited chondrocyte glycolysis and OA cartilage proliferation, suggesting that PKM2 can regulate glycolysis and ECM production, which provides new insights into PKM2 as a potential target for the treatment of OA.

### 3.5 Role of PFKFB3-mediated glucose metabolism in OA

Fructose 2, 6-diphosphate (F2,6BP) is one of the important regulators of glycolysis and an allosteric activator of phosphofructokinase-1 (PFK-1). F2,6BP is mainly synthesized and degraded by 6-phosphofructose-2-kinase/fructose 2, 6-bisphosphatase (PFKFB1/2/3/4). Among them, PFKFB3 has a high kinase/phosphatase activity, therefore, it is the most widely expressed in the human tissues, and is also the main studied isomerase (66). At present, it has been confirmed that PFKFB3 can affect the physiological activities of various tissues. Interestingly, PFKFB3 expression can regulate glycolysis in tumor cells and cancer cells (67, 68). In addition, inhibition of PFKFB3 was found to be associated with decreased insulin-stimulated glucose uptake, GLUT4 translocation, AKT signaling, and glycolytic flux in cancer cells and adipocytes (69). Based on this, PFKFB3 may play a role in OA cartilage and its dysfunction may participate in the pathogenesis of OA. Data from Qu et al. showed that PFKFB3 expression was decreased in OA cartilage tissue as well as tumor necrosis factor (TNF)-α- or IL-1β-regulated chondrocytes, accompanied by decreased glucose utilization, ATP production, and lactate production, suggesting that PFKFB3 is impaired in OA and contributes to glycolysis disorders (70). Similarly, inhibition of PFKFB3 in FLS cells from synovium of patients with inflammatory arthritis correlated with decreased glucose uptake and lactate production (54, 71). More importantly, PFKFB3 was reported to enhance the cell viability of chondrocytes through the PI3K/AKT/C/EBP homologous protein (CHOP) signaling pathway (70). It is also capable of reducing caspase 3 activation and promoting aggrecan and type II collagen expression in OA cartilage explants and chondrocytes, possessing potential to be a target for OA prevention and treatment.

### 3.6 Role of AMPK-mediated glucose metabolism in OA

AMP-activated protein kinase (AMPK) is a major regulator of energy metabolism and balance, possessing the potential to be a therapeutic target for metabolic disorders and aging-related diseases, including OA (7, 72, 73). A reduction in AMPK activity was found in both human and mouse knee OA cartilage as assessed by phosphorylation of specific threonines in AMPKα1, the AMPK-activated α subunit, suggesting that AMPK activity in chondrocytes plays an important role in maintaining joint function stability and the development of OA (74). Indeed, Zhou et al. (75) found in a mouse model that using cartilage-specific, tamoxifen-inducible AMPKα knockout mice exhibited accelerated severity of surgically induced OA compared to wild-type mice, and at 12 months, male mice developed severe spontaneous aging-related OA lesions. These results demonstrate that chondrocyte-specific AMPKα depletion in adulthood leads to accelerated OA progression (29, 74). Notably, the activation of AMPK is partially achieved by maintaining glycolysis. Specifically, OA-associated degradation features in AC are a downstream consequence of altered metabolism of resident chondrocytes. These OA features include enhanced production of extracellular proteases such as MMPs. Ohashi et al. (76) found that chondrocytes were more dependent on glycolysis as a source of cellular ATP after il-1β treatment, while only a small fraction was derived from OXPHOS. Blocking IL-1β-enhanced MMP13 levels using galactose substitution (a known activator of OXPHOS and inhibitor of glycolysis) in OA chondrocytes led to a reverse increase in phospho-AMPK-related biomarkers, confirming AMPK as a downstream regulator during OA glycolysis.

Current studies have confirmed the important role of AMPK and aerobic glycolysis in the pathogenesis of OA and provided clues for further exploration.

**Figure 1:**
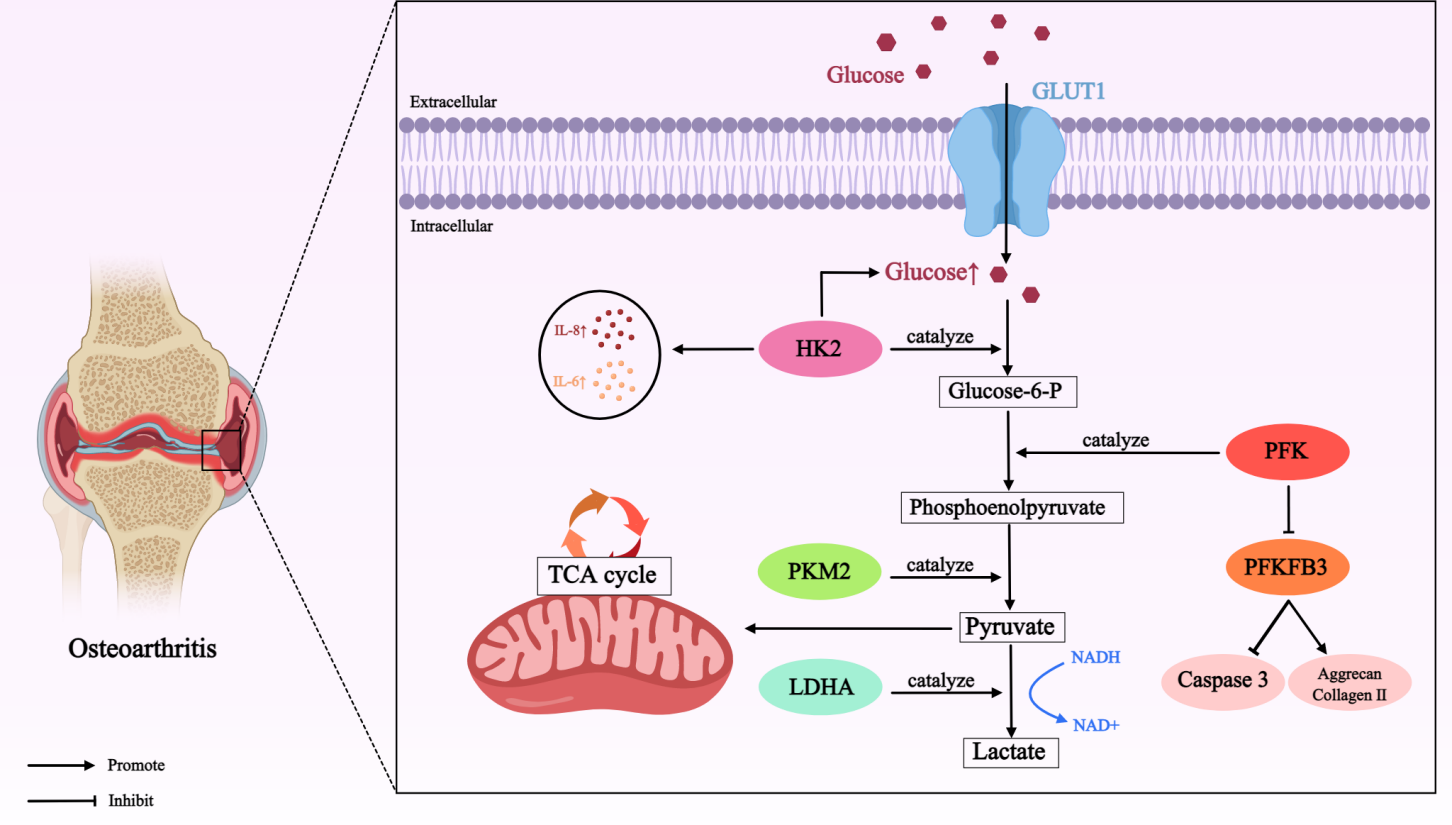
Role of glucose metabolism mediated by different enzymes in OA. In OA cartilage, glucose is transported by GLUT1 (a passive transporter that relies on the concentration gradient as the driving force for the movement of glucose molecules across the membrane) into chondrocytes for metabolism. Many enzymes involved in glucose metabolism, such as HK2, PKM2, LDHA, PFK, PFKFB3, initiate the major pathways of glucose utilization (including glycolysis) in this process, thereby directly or indirectly affecting the development of OA. Glucose transporter type 1 (GLUT1); hexokinase 2 (HK2); interleukin-8 (IL-8); interleukin-6 (IL-6); phosphofructokinase (PFK); 6-phosphofructose-2-kinase/fructose 2, 6-bisphosphatase 3 (PFKFB3); pyruvate kinase M2 (PKM2); lactate dehydrogenase A (LDHA); nicotinamide-adenine dinucleotide (NADH).

All in all, the role of glucose metabolism mediated by different enzymes in OA is shown in **Table 1** and **Figure 1**.

**Table 1:** The role of targeted regulation of glucose metabolism in OA.

Regulator	Role in glucose metabolism	Mechanisms of action in OA (Cell source)	References
Role of glucose metabolism mediated by different enzymes in OA			
GLUT1	Glucose uptake	1. Enhance glucose uptake, balance glucose levels in cells, and produce excess AGEs (human OA chondrocytes); 2. The loss of *GLUT1* gene leads to GP cartilage, articular cartilage abnormalities, cytopenia and proteoglycan loss in articular cartilage (mice chondrocytes)	(16, 22–28)
HK2	Initiate major glucose utilization glycolysis, pentose phosphate pathway, and OXPHOS; convert glucose to G6P	1. Promote glucose entry into the cell; 2. Lead to increased RNA expression levels of proinflammatory cytokines (human OA FLS); 3. Gene expression of *HK2* was increased in severe OA cartilage (human primary chondrocytes)	(17, 19, 29–31)
LDHA	Key enzyme in aerobic glycolysis, catalyze the conversion of pyruvate into lactate and ROS production	1. Inhibit lactate secretion and promoted HAS2 and HA synthesis (SFs from TMJOA patients); 2. mediate high-throughput glycolysis	(9, 20, 32)
PKM2	Rate-limiting enzyme in glycolysis; catalyze PEP to pyruvate and ATP	1. PKM2 knockout inhibits glycolysis and reduces the production of extracellular collagen matrix; increase lactate accumulation; inhibit chondrocyte matrix synthesis and promotes OA cartilage degeneration (human OA chondrocytes)	(38–40)
PFKFB3	Regulators of glycolysis	1. Contribute to glycolysis disorders (human OA cartilage); 2. decrease glucose uptake and lactate production (FLS cells from synovium of patients with arthritis)	(41–45)
AMPK	Major regulator of energy metabolism and balance	1. A downstream regulator during OA glycolysis (human OA chondrocytes)	(19, 49, 50)
Role of glucose metabolism mediated by different factors in OA			
HIF-1α	Induce a switch from oxidative to glycolytic metabolism	1. Increase glucose uptake and utilization to promote anaerobic ATP production (human OA chondrocytes); 2. increase the expression of glycolytic enzymes; increase the expression of GLUT1, G6PD, PFK1, and PDK1 (mice primary articular chondrocytes)	(52–59)
TGF-β1	Inhibit glycolysis; induce OXPHOS conversion to aerobic glycolysis; maintain chondrocyte homeostasis and articular cartilage integrity	1. Increase glucose consumption and lactate production, as well as a decrease in ATP levels; elevate the expression of key glycolytic enzymes such as GLUT1 and HK2 to increase glycolysis and lactate production (human OA chondrocytes)	(7, 17, 62, 63, 65)
NF-κB	Increase the production of downstream cytokines	1. Increase the expression of cartilage degradation genes and promote chondrocytes dysfunction; inflammation can shift cellular metabolism toward NF-κB-mediated aerobic glycolysis (mice chondrocytes)	(9, 66–69)
Potential therapeutic substances for OA that affect glucose metabolism			
Icariin	An inducer of HIF-1α and an activator of glucose metabolism in chondrocytes	1. Promote the up-regulation of HIF-1α and PDK1 expression in chondrocytes, leading to the increased expression of key targets (G6PD and PGK1) and the inhibition of mitochondrial OXPHOS, ultimately inducing glucose metabolism in a glycolytic manner; promote the expression of GLUT1 and other glycolytic enzymes; promote anaerobic glycolysis and increased cell viability in OA chondrocytes (mice chondrocyte)	(58, 70–72)
2-deoxy-D-glucose	An inhibitor of glycolysis with the potential to affect glucose metabolism	1. Altered glucose use and reduced lactate production in an inflammatory environment (human OA chondrocytes)	(1, 19, 73, 74)
Glucocorticoids	Shift carbohydrate metabolism from mitochondrial respiration to glycolysis	1. Anti-inflammatory; regulate glucose and lipid metabolism; up-regulate PDK4 (human OA chondrocytes); 2. affect IGF1 and GLUT1 in cartilage tissue through GC-IGF1-GLUT1 axis (mice chondrocyte)	(28, 75–78)
Galactose	Galactose substitution has been proposed as an OXPHOS activator with the effect of inhibiting glycolysis to produce ATP	1. Enhance chondrocyte dependence on the glycolytic pathway for ATP production (bovine chondrocytes)	(50, 63, 79)
Glucose	Provide adequate glucose to maintain the major anaerobic metabolism of articular chondrocytes	1. Affect the balance of matrix synthesis and degradation; affect glucose uptake as well as matrix production and maintenance, and promote the anti-inflammatory activity of differentiated chondrocytes (equine chondrocytes)	(80, 81)

OA, osteoarthritis; GLUT, glucose transporter; AGEs, advanced glycation end products; HK2, hexokinase 2; OXPHOS, oxidative phosphorylation; G6P, glucose-6-phosphate; FLS, fibroblast-like synoviocytes; LDHA, lactate dehydrogenase A; ROS, reactive oxygen species; HAS2, hyaluronic acid synthetase 2; HA, hyaluronic acid; TMJOA, temporomandibular joint osteoarthritis; SFs, synovial fibroblasts; PKM2, pyruvate kinase M2; PEP, phosphoenolpyruvate; PFKFB3, 6-phosphofructose-2-kinase/fructose 2, 6-bisphosphatase 3; AMPK, AMP-activated protein kinase; HIF-1α, hypoxia-inducible factor 1α; G6PD, glucose-6-phosphate dehydrogenase; PFK1, phosphoglycerate kinase 1; PDK1, pyruvate dehydrogenase kinase 1; TGF-β1, transforming growth factor-β1; NF-κB, nuclear factor kappa B; PGK1, phosphoglycerate kinase 1; PDK4, pyruvate dehydrogenase kinase 4; IGF1, insulin-like growth factor-1.

## 4 Role of glucose metabolism mediated by different factors in OA

### 4.1 Role of HIF-1α-mediated glucose metabolism in OA

HIF-1 is a highly conserved transcription factor and a major regulator of cellular adaptation to hypoxic stimulation (77). The activity of HIF-1 is primarily dependent on its alpha subunit (HIF-1α) (78). Because cartilage is relatively thick, chondrocytes at the surface of the joint are exposed to only about 6% to 10% of oxygen, while the deepest chondrocytes have access to even less oxygen in the cartilage (79). In hypoxic cells, HIF-1α can induce a switch from oxidative to glycolytic metabolism. Moreover, it is of critical importance in maintaining chondrocytes activity and integrity, cartilage energy production, and regulating chondrogenesis, energy metabolism, and matrix synthesis (80, 81). Studies have shown that the treatment of OA can significantly down-regulate the expression of *HIF-1α* gene, suggesting that HIF-1α plays an important role in the pathological mechanism of OA (82). Yudoh et al. (83) revealed that OA chondrocytes lacking HIF-1α fail to produce energy and maintain ECM growth under both normoxic and hypoxic conditions, suggesting that chondrocytes may regulate ATP levels and ECM production during OA through adaptive functions of the *HIF-1α* gene. Metabolically, activated OA chondrocytes rely on HIF-1 to increase glucose uptake and utilization to promote anaerobic ATP production, thereby compensating for the accelerated energy expenditure during OA (84). HIF-1α also increases the expression of glycolytic enzymes, such as HIF-1α augments the expression of phosphoglycerate kinase, which converts 1, 3-diphosphoglycerate to 3-phosphoglycerate during glycolysis (85). Besides, under the influence of the complex of HIF-1α, HIF-1β and HREs, the expression of GLUT1, G6P dehydro-genase (G6PD), phosphoglycerate kinase 1 (PGK1) and pyruvate dehydrogenase kinase 1 (PDK1) is increased, which leads to promoting glucose transfer and anaerobic glycolysis (79, 86). Furthermore, vascular endothelial growth factor (VEGF) and erythropoietin (EPO) have been reported to increase oxygen and nutrient delivery, as well as metabolic adaptations, thereby preventing chondrocyte death in the growth plate. Under hypoxia, HIF-1α promotes glycolysis by inducing the expression of both (87). Therefore, further studies on the role of HIF-1α in OA and its potential mechanism may provide a new therapeutic target for the treatment of OA.

### 4.2 Role of TGF-β1-mediated glucose metabolism in OA

Transforming growth factor (TGF)-β1 is a multifunctional cell regulatory factor, mainly involved in the process of growth and development, proliferation and differentiation, ECM synthesis and immune response (88, 89). Recent studies have indicated that TGF-β1 can inhibit glycolysis by inducing natural regulatory T cells and down-regulating the expression of critical enzymes of glycolysis such as GLUT1 and HK2 (29, 90). TGF-β1 stimulation can promote the glycolytic process in dermal fibroblasts, while inhibition of glycolysis is able to impede TGF-β1-induced profibrosis, suggesting that TGF-β1 plays a dominant role in inducing OXPHOS conversion to aerobic glycolysis (91). Additionally, accumulating evidence supports that the maintenance of AC morphology and the intracellular environment of chondrocytes relies on the regulatory function of TGF-β1 (16, 21). Wang et al. (16) found an increase in glucose consumption and lactate production, as well as a decrease in ATP levels, in OA chondrocytes from humans treated with TGF-β1. Moreover, TGF-β1 stimulation significantly elevated the expression of key glycolytic enzymes such as GLUT 1 and HK2 to increase glycolysis and lactate production. Interestingly, HK1 was also found to be upregulated under TGF-β1 treatment (16). Likewise, Ni et al. detected that TGF-β1 increased HK1 and HK2 expression and simultaneously upregulated HK activity in c-Kit cell lines, suggesting that TGF-β1 has the potential to directly regulate HK and providing important clues for TGF-β1-mediated OA pathogenesis (92). However, the current therapeutic potential of TGF-β1 in OA is still controversial, as it plays both protective and detrimental roles in OA. Therefore, the molecular mechanisms of glucose metabolism in OA targeting TGF-β1 deserve further attention to provide insights into the regulation of energy metabolism and lead to exploring new therapeutic approaches for effective management of OA by modulating cellular metabolism.

### 4.3 Role of NF-κB -mediated glucose metabolism in OA

Nuclear factor kappa B (NF-κB) is a transcription factor that widely presents in a variety of cell lines and participates in various cellular inflammatory responses (93). Mechanistically, in chondrocytes, NF-κB activation may play an important role in the progression of OA by increasing the expression of cartilage degradation genes and promoting chondrocytes dysfunction (18). An earlier study found that NF-κB activation is involved in regulating cartilage differentiation (94). In addition, Sang et al. (95) found that glycolysis and microenvironment remodeling could be promoted by activating the Ca^2+^/NF-κB axis to increase the production of downstream cytokines, suggesting that the role played by NF-κB signaling is critical in the regulation of glycolytic activity. In addition, Wang et al. (96) reported a positive correlation between HK2 expression and inflammatory cell infiltration and cartilage destruction in arthritic rats treated with glycolysis inhibitors, implying that HK2 glycolysis inhibitors are closely related to NF-κB activation and inhibition. Furthermore, Arra et al. (18) showed that inflammation can shift cellular metabolism toward aerobic glycolysis mediated by NF-κB, further confirming the strong links between inflammatory and metabolic responses in OA chondrocytes. The above evidence clearly establishes that NF-κB activation can mediate glycolytic process to drive OA pathological changes, but further studies are needed to reveal the precise association and underlying mechanism.

**Figure 2:**
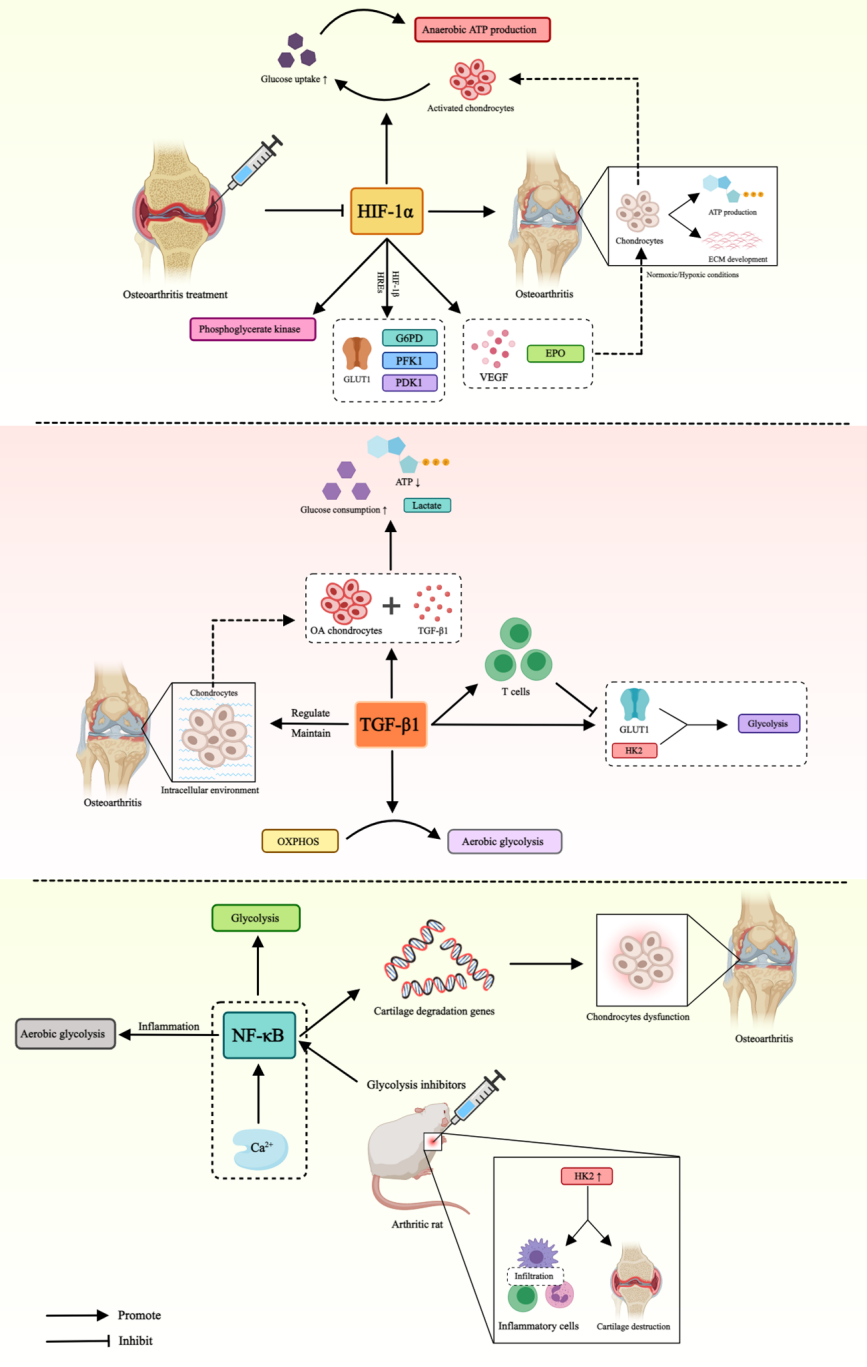
Role of glucose metabolism mediated by different factors in OA. This figure illustrates that HIF-1α, TGF-β and NF-κB affect the pathogenesis of OA by mediating different pathways of glucose metabolism. Hypoxia-inducible factor-1α (HIF-1α), glucose transporter type 1 (GLUT1), hypoxia-inducible factor-β1 (HIF-β1), hypoxia-responsive elements (HREs), glucose-6-phosphate dehydro-genase (G6PD), phosphoglycerate kinase 1 (PFK1), pyruvate dehydrogenase kinase 1 (PDK1), vascular endothelial-derived growth factor (VEGF), erythropoietin (EPO), transforming growth factor-β1 (TGF-β), hexokinase 2 (HK2), oxidative phosphorylation (OXPHOS), nuclear factor kappa B (NF-κB).

In conclusion, the role of glucose metabolism mediated by different factors in OA is presented in **Table 1** and **Figure 2**.

## 5 Potential therapeutic substances for OA that affect glucose metabolism

Targeting glucose metabolism switches, especially glucose transporters and regulatory factors involved in mediating glucose metabolism, is challenging but has potential therapeutic implications in OA. The following substances (summarized in **Table 1**) play an important role in inhibiting OA development by affecting glucose metabolism.

### 5.1 Icariin

Icariin (ICA), a flavonoid compound extracted from Herba Epimedii (HEP), is known to be an inducer of HIF-1α and an activator of glucose metabolism in chondrocytes (86, 97). As reported by Wang et al. (86) in chondrocyte experiments in mice, ICA treatment upregulated the expression of SOX9, a key ECM regulator that contributes to the chondrocyte phenotype, suggesting that ICA can promote chondrocyte viability and formation. Specifically, as a glucose carrier and a downstream target of HIF-1α, GLUT1 is activated after ICA treatment. Similarly, ICA treatment also enhanced the expression of PGK1, which is both a critical enzyme in anaerobic glycolysis and a downstream target of HIF-1α (86, 98). PDK1 is capable of limiting oxygen consumption by preventing TCA cycle, thereby promoting anaerobic glycolysis. Additionally, ICA has been shown to up-regulate the expression of G6PD, which is a rate-limiting enzyme in the pentose phosphate pathway and an important regulator of energy expenditure and glucose metabolism. Mechanistically, ICA treatment promoted the up-regulation of HIF-1α and PDK1 expression in chondrocytes, leading to the increased expression of key targets (G6PD and PGK1) and the inhibition of mitochondrial OXPHOS, ultimately inducing glucose metabolism in a glycolytic manner (86). To sum up, ICA treatment promoted the expression of GLUT1 and other glycolytic enzymes, which contributed to anaerobic glycolysis and increased cell viability in OA chondrocytes (99). Therefore, ICA is expected to represent a novel treatment for OA.

### 5.2 2-deoxy-D-glucose

2-deoxy-D-glucose (2-DG) is widely known as an inhibitor of glycolysis with the potential to affect glucose metabolism (100). Ying et al. (6) demonstrated that glucose metabolism is an inflammatory target for OA progression *in vivo* by intraperitoneal injection of 2-DG. The results suggest that 2-DG attenuates the development of OA in mice, and the underlying mechanism may be related to the inhibition of subchondral bone sclerosis and cartilage degeneration. Meanwhile, they also found that 2-DG had a protective effect on destabilization of the medial meniscus surgery (DMM)-induced OA mice by inhibiting glycolysis. Previous studies have recommended an effective dose of 2-DG to sufficiently inhibit glycolytic activity in animals, but it remains important to optimize this dose to prevent cartilage damage in OA (101). In addition, Wu et al. (31) found in primary chondrocytes collected from patients undergoing total knee arthroplasty surgery that altered glucose use and reduced lactate production in an inflammatory environment have been proposed as potential mechanisms by which 2-DG inhibits the expression of cartilage catabolic genes by suppressing glycolysis. Terabe et al. (102) also demonstrated that 2DG is a potent blocker of the glycolytic pathway in chondrocytes in IL1β-induced human OA chondrocytes and bovine chondrocytes. Overall, the glycolytic inhibitor 2-DG provides an experimental therapeutic option for OA, but further studies are still needed to observe the potential effects of 2-DG on chondrogenesis and overall anabolic activity.

### 5.3 Glucocorticoids

Glucocorticoids (GCs) are steroid hormones produced endogenously by the adrenal cortex. In target cells, they bind to glucocorticoid receptors (GR) to form complexes and then migrate into the nucleus (103, 104). Clinically, intra-articular injection of GCs is widely used to relieve various types of inflammation and pain (103, 105). There are two potential mechanisms underlying the anti-inflammatory effects: 1) enhancing the expression of anti-inflammatory genes through the binding of GR-steroid complexes to glucocorticoid response elements (GREs); 2) inhibiting the activity of inflammatory transcription factors such as NF-κB and activator protein 1 (AP-1) (103). More importantly, GCs are also recommended for the treatment of knee OA. In OA chondrocytes, in addition to their anti-inflammatory effects, GCs also partially mediates their effects on OA cartilage by regulating glucose and lipid metabolism. Interestingly, *pyruvate dehydrogenase kinase 4 (PDK4)* was one of the most significantly up-regulated genes by dexamethasone (a glucocorticoid). Since this gene is able to inhibit pyruvate dehydrogenase activity and prevent the progression of pyruvate produced by glycolysis to OXPHOS, the role of GCs may be to shift carbohydrate metabolism from mitochondrial respiration to glycolysis (106). Furthermore, Li et al. (44) reported that GCs could affect IGF1 and GLUT1 in cartilage tissue through the GC-IGF1-GLUT1 axis. In detail, IGF1 can promote the up-regulation of GLUT1 expression, leading to the accumulation of AGEs in chondrocytes, thereby promoting the inflammation and matrix degradation of AC, and ultimately inducing OA. Therefore, future studies to further elucidate the role of GCs in glucose metabolism associated with OA pathophysiology may be an interesting avenue to investigate.

### 5.4 Galactose

Galactose substitution has been proposed as an OXPHOS activator with the effect of inhibiting glycolysis to produce ATP (91). In addition, galactose substitution is a common method to study the downstream effects of metabolic function in cancer or normally proliferating cells. Typically, G6P converted from galactose is involved in the glycolytic pathway, and the conversion rate of galactose to G6P is slower than that of glucose (107). Thus, pyruvate produced through the glycolytic metabolism of galactose produces less ATP, forcing the cell to increase its dependence on ATP produced by OXPHOS. Ohashi et al. (76) observed in bovine and human primary articular chondrocytes a strong dependence of chondrocytes on ATP produced by the glycolytic pathway in glucose-rich medium, which became more prominent upon stimulation with IL-1β and, in turn, echoed the absence of ATP produced by OXPHOS. In contrast, chondrocytes in galactose-substituted medium showed a significant reduction in glycolysis and an increase in ATP produced by mitochondrial activity due to the enhanced overall mitochondrial function, suggesting that repairing mitochondrial dysfunction may be one of the effective strategies for the treatment of OA by targeting catabolism. Similarly, Lane et al. (108) found significant reductions in lactate production and maximal lactate dehydrogenase activity in healthy bovine primary chondrocytes with increasing time of galactose culture, suggesting inhibition of chondrocyte glycolysis and decreased dependence of cellular ATP production on the glycolytic pathway, confirming the ability of galactose culture to alter chondrocyte metabolism.

### 5.5 Glucose

Glucose plays an essential role in the maintenance of chondrocyte metabolism, and adequate glucose supply is required to maintain the major anaerobic metabolism of articular chondrocytes. In addition, glucose is also a key precursor for the synthesis of ECM macromolecules by these cells (109). Therefore, inflammatory diseases such as OA may have a higher glucose requirement, but the demand for chondrocytes is limited due to the avascular nature of cartilage tissue. It should be emphasized that limited glucose supply carries the risk of impairing cellular function and may lead to an imbalance in matrix synthesis and degradation, resulting in OA (56). Of interest, Sopasakis et al. (56) investigated the pivotal role of glucose as a fuel source for matrix generation and cartilage repair in conditions of OA using differentiated equine chondrocytes pellets in a 3D model *in vitro*. The results showed that in the presence of inflammatory conditions associated with OA, heightened glucose levels not only facilitate glucose uptake, HA synthesis and enhance matrix production and maintenance, but also promote the anti-inflammatory activity of differentiated chondrocytes. This indicates that sufficient glucose supply to chondrocytes promotes cartilage repair by interrupting the harmful inflammatory cycle and inducing the synthesis of HA, suggesting that intra-articular injection of glucose is beneficial to the early treatment of OA, and its combination with other therapies is expected to improve the therapeutic effect by regulating chondrocytes metabolism and inducing anti-inflammatory processes. Notably, a large number of studies have shown that both diabetes mellitus and type 2 diabetes mellitus are significantly associated with OA, and diabetic patients have a higher risk of OA, suggesting that hyperglycemia may induce or aggravate OA (110, 111). Li et al. (112) demonstrated in high glucose-stimulated rat FLS in the *in vitro* cocultivation system that hyperglycemia leads to the accumulation of AGEs in FLS through the HIF-1α-GLUT1 pathway, thereby increasing the release of FLS inflammatory factors, which in turn induces chondrocyte degradation and promotes OA development. Of note, after being activated by AGEs, the receptor of AGEs (RAGE) can trigger downstream inflammatory signals to participate in a variety of degenerative diseases. Therefore, RAGE inhibitors are expected to block the proinflammatory effects of high glucose.

Prolotherapy is a safe nonsurgical regenerative injection technique that stimulates the production of growth factors and cytokines, and promotes growth of normal cell and tissue by injecting small amounts of a stimulating solution (including hypertonic dextrose, morrhuate sodium, dextrose/phenol/glycerin solution, or platelet-rich plasma) into painful and degenerative joints, ligaments, and adjacent joint spaces, leading to the regeneration of previously damaged ligaments, tendons, and other intra-articular structures (113). A previous systematic review suggests that dextrose prolotherapy may benefit patients with knee OA by promoting tissue repair and improving range of motion and joint stability. OA patients treated with dextrose showed significant improvement compared to those receiving saline injection and normal treatment (114). In addition, another systematic review including 10 articles indicated that dextrose prolotherapy has been shown to have better therapeutic effects on pain in patients with OA compared to exercise, and that it has similar effects to platelet-rich plasma and steroid injections (115). However, although the latest systematic review showed that dextrose prolotherapy is beneficial in reducing pain and improving function in patients with knee OA, there is a high risk of bias and heterogeneity in the results (116, 117). While individuals may experience short-term relief, questions remain about the long-term durability of the treatment. This uncertainty can make it challenging to assess the suitability of treatment for sustained management of knee OA. Additionally, the dextrose concentration/volume, interval and duration of treatment, as well as injection site and technique may differ. The absence of standardized treatment protocols for dextrose prolotherapy contributes to variability in how the procedure is performed. Therefore, despite the promising results, there is still a need for larger clinical trials with a standardized treatment regimen and long-term findings to show improvement in the quality of life consistently.

## 6 Conclusions

In conclusion, improving the glucose metabolism is conducive to treating OA, mainly through regulating the glycolysis. Due to the hypoxic environment, chondrocytes are highly dependent on glycolysis, which is the main source of energy for chondrocytes. Thus, in OA chondrocytes, the regulation of glucose transport and glycolytic pathways is thought to influence the pathogenesis of the disease, and the process involves the responses of multiple enzymes. Among them, GLUT1, HK2, LDHA, PKM2, PFKFB3, and AMPK are closely related to cell growth, proliferation, metabolism, and apoptosis, and play a vital role in the development of OA mainly by affecting the glycolytic process. In addition, many studies have reported key factors in the process of glucose metabolism, suggesting their involvement in the pathogenesis of OA. This review emphasizes the potential mechanisms of HIF-1α, TGF-β1, NF-κB and their regulation pathway in energy metabolism and progression of OA. They are also important targets for the regulation of glycolysis. More importantly, ICA, 2-DG, GC, galactose and glucose have been shown to inhibit OA by regulating glucose metabolism, which provides new insights into the treatment of OA. However, different administration methods, concentrations and dosages still need to be further explored to ensure the best results.

## 7 Future perspectives

Energy metabolism is central to the maintenance of cartilage function. Accumulating studies are interested in the role of glucose metabolism in the pathogenesis and therapeutic mechanisms of OA. In an adverse microenvironment, energy conversion to glycolysis-dominated glucose metabolism is critical for immune response and inflammatory pathway activation during OA progression. From a translation perspective, the pathogenesis of OA is considered to be related to differences in glucose utilization between normal and diseased chondrocytes (16, 31). Targeting glucose transporters and rate-limiting enzymes during glucose metabolism may be an important strategy for the treatment of OA. However, future studies are needed to further confirm the comprehensive details of the specific regulatory mechanisms and signaling pathways. In addition, studies on the key factors affecting glucose metabolism such as HIF-1α, TGF-β1, NF-κB, and their mediated signaling pathways are expected to further reveal the potential mechanisms regulating energy metabolism in OA, which will contribute to developing small molecule drugs targeting the downstream proteins or kinases. Furthermore, metabolomics techniques have suggested some potential metabolic pathway alterations in OA. Future research of OA should focus on specific phenotypes and related molecular pathways. The disease-related alterations of specific components and regulatory molecules in glucose metabolism pathways such as glycolysis, TCA cycle, and their interactions in OA progression warrant further investigation. From a therapeutic perspective, it will also be critical to explore the potential of glucose metabolism shifts in current effective OA therapies, such as physical exercise and weight loss, and the response to experimental drugs that affect cellular glucose metabolism. Similarly, immunometabolism should also be used as a key means of understanding OA pathophysiology in the future.

## Author contributions

PP: Writing – original draft, Writing – review & editing. LZ: Writing – original draft, Writing – review & editing. ZZ: Writing – review & editing. KZ: Writing – review & editing. BH: Writing – review & editing. XB: Writing – review & editing. YW: Conceptualization, Writing – review & editing.
